# Genetic and Respiratory Pathogenic Comparison of Two PRRSV‐2 Isolates Classified as Lineage Korean A and B

**DOI:** 10.1155/tbed/3335031

**Published:** 2026-04-15

**Authors:** Hyejin Na, Chanhee Chae

**Affiliations:** ^1^ Department of Veterinary Pathology, College of Veterinary Medicine, Seoul National University, Seoul, 08826, Republic of Korea, snu.ac.kr

## Abstract

Porcine reproductive and respiratory syndrome virus (PRRSV) continues to cause substantial economic losses worldwide, and certain lineages circulate in specific countries. Lineage Korean A (LKA), B (LKB), and C (LKC) have been reported exclusively in Korea. However, studies on LKA and LKB have been limited to strains isolated before 2015. In this study, we analyzed the genetic and pathogenic characteristics of SNUVP2403A and SNUVP2407B strains isolated in 2024. Based on ORF5 phylogenetic analysis, SNUVP2403A and SNUVP2407B were classified as LKA and LKB, respectively. No recombination was detected in SNUVP2403A, whereas SNUVP2407B was identified as a recombinant between NADC30 and JB15‐N‐PJ10‐GN. In the animal infection study, the pigs inoculated with SNUVP2403A had a mortality rate of 8.3% (1/12 pigs), and clinical signs such as nasal discharge, coughing, eyelid edema, and cyanosis were observed. The group inoculated with SNUVP2407B exhibited only mild respiratory symptoms, and all pigs survived throughout the experiment. Therefore, it was demonstrated that SNUVP2403A is more virulent than SNUVP2407B. In addition, SNUVP2407B exhibited significantly lower pathogenicity than previously reported LKB strains. In conclusion, this study provides a systematic characterization of SNUVP2403A and SNUVP2407B and improves our understanding of the genetic diversity and pathogenicity of PRRSV circulating in Korea.

## 1. Introduction

Porcine reproductive and respiratory syndrome (PRRS) is a prevalent viral disease in the global swine industry [1, 2]. PRRS is characterized by respiratory disease in pigs of all ages and reproductive failure in sows [3, 4]. The etiologic agent, PRRS virus (PRRSV), belongs to the family *Arteriviridae* and the order *Nidovirales* [5, 6]. PRRSV is an enveloped virus and its genome is a non‐segmented, positive‐sense single‐stranded RNA of ~15 kb in length. It consists of a 5^′^ untranslated region (UTR), at least 11 open reading frames (ORFs), a 3^′^ UTR, and a poly(A) tail [7, 8]. Currently, PRRSV exists as two distinct species, *Betaarterivirus europensis* (PRRSV‐1) and *Betaarterivirus americense* (PRRSV‐2) [9].

PRRSV is one of the viruses in which mutation and recombination occur most frequently, resulting in high genetic diversity [10, 11]. In particular, ORF5 is the most genetically diverse region in the genome of PRRSV and encodes glycoprotein 5, which is the target of neutralizing antibodies [12–14]. Accordingly, phylogenetic analysis based on ORF5 sequences defines topologically distinct monophyletic clusters as lineages, within which genetic divergence is ≤11%. In addition, clusters showing more than 7% genetic divergence within a lineage are classified as sublineages. Based on these criteria, PRRSV‐2 was divided into 9 lineages and 37 sublineages in 2010 [15]. Subsequently, three phylogenetic clusters were identified exclusively in Korea. They were designated as lineage Korea A (LKA), B (LKB), and C (LKC) [16]. Recently, the PRRSV‐2 classification has been refined into 11 lineages (L1–L11) and 21 sublineages (L1A–L1F, L1H–L1J, L5A–L5B, L8A–L8E, and L9A–L9E), with LKA and LKC reassigned to L11 and L1J, respectively [17]. Meanwhile, LKB was proposed as an additional novel lineage (L12), as it exhibits greater than 15% ORF5 genetic divergence from other established PRRSV‐2 lineages [18].

Genetic and pathogenic studies have been conducted on LKA and LKB strains isolated before 2015 and LKC strain isolated in 2023 [19]. Whole genome analysis revealed that LKA (GBGJ22) and LKB (GGYC45) strains are recombinant viruses derived from LKC as the major parental strain and a modified live vaccine strain (L5) as the minor parent [20]. Animal studies demonstrated that LKA strains (GBGJ22 and CBJE19) were moderately pathogenic and LKB strains (GGYC45 and JB15‐N‐PJ10‐GN) were highly pathogenic [19], highlighting the need for ongoing surveillance of these lineages. In this study, we investigated the genetic and pathogenic characteristics of two field isolates classified as LKA and LKB based on ORF5 phylogenetic analysis, which were obtained from Korean farms in 2024.

## 2. Materials and Methods

### 2.1. Farm History

In January 2024, respiratory symptoms and fever occurred in weaned piglets on a farm located in Chungnam Province, Korea, and the mortality rate reached 20%. In July 2024, another farm in Gyeongbuk Province reported that 70‐day‐old pigs coughed for 1 week.

### 2.2. Virus Isolation and Propagation

Blood samples and lung tissues were obtained from both farms. Blood samples were centrifuged at 2000 rpm for 10 min to obtain serum. Lung tissues were homogenized in Dulbecco’s Phosphate‐Buffered Saline (D‐PBS) and centrifuged at 2500 rpm for 10 min. The serum and lung supernatants were filtered using a sterile 0.2 μm syringe filter.

Pulmonary alveolar macrophages (PAMs) were obtained from 3‐week‐old pigs by lung lavage. Pigs were confirmed negative for PRRSV, porcine circovirus, swine influenza virus, and Mycoplasma by real‐time PCR. PAMs were cultured in RPMI‐1640 medium supplemented with 10% fetal bovine serum (FBS) and 1% antibiotic–antimycotic at 37°C with 5% CO_2_. African green monkey kidney (MARC‐145) cells were cultured in Dulbecco’s Modified Eagle’s Medium (DMEM) containing 5% FBS and 1% antibiotic–antimycotic at 37°C with 5% CO_2_. The filtered samples were diluted with growth medium and inoculated into PAMs and MARC‐145 cells at ~80% confluence. After 1 h of inoculation with shaking every 15 min, the cells were washed with D‐PBS.

Inoculated PAMs and MARC‐145 cells were harvested by three cycles of freezing and thawing. After the third passage, the cultures were harvested for immunofluorescence assay (IFA), immunocytochemistry (ICC), and as inocula for the animal experiment. The inocula were filtered through a 0.2 μm syringe filter. The third‐passaged cultures for whole genome sequencing (WGS) were harvested using a cell scraper without freezing and thawing.

### 2.3. IFA

The virus was diluted 10‐fold and inoculated into PAMs and MARC‐145 cells for IFA. The cells were fixed with 80% acetone for 15 min. Normal goat serum (Vector Laboratories, Burlingame, CA, USA) was applied for 1 h to block non‐specific antibody binding. The cells were incubated with the PRRSV nucleocapsid‐specific monoclonal antibody SR‐30 (Rural Technologies Inc., Brookings, SD, USA), diluted 1:1000. Alexa Fluor 488 (Thermo Fisher Scientific, Waltham, MA, USA) diluted 1:1000 was applied. Nuclear staining was performed using 4’,6‐diamidino‐2‐phenylindole (DAPI), and fluorescent images were acquired using a THUNDER Imager Live Cell & 3D Assay (Leica Microsystems, Wetzlar, Germany). Based on the IFA results, the 50% tissue culture infectious dose (TCID_50_/mL) of both viruses was calculated. The third passage viruses were diluted to 0.01 MOI for growth curve analysis and to 10^5^ TCID_50_/mL for the animal experiment.

### 2.4. Determination of Multi‐Step Growth Curve

The diluted viruses were inoculated into PAMs, and culture supernatants were collected at 0, 12, 24, 36, and 48 h post‐inoculation (hpi). Viral genomic copy numbers were quantified using one‐step real‐time PCR. In addition, ICC was performed to determine the TCID_50_/mL of the supernatants collected at each time point. The procedure was identical to that of IFA up to the primary antibody binding step. Goat anti‐mouse antibody was used as the secondary antibody, and Vector Red AP Substrate Kit (Vector Laboratories, Newark, CA, USA) was used as the chromogenic substrate.

### 2.5. WGS

The viruses from the third passage were used for next‐generation sequencing (NGS) as previously reported in our laboratory [21, 22]. RNA was extracted from harvested cell cultures with the Direct‐zol RNA Miniprep Kit (Zymo Research, Irvine, CA), and NGS was performed on the extracted RNA by Macrogen Inc. (Seoul, Korea). cDNA libraries were prepared using the TruSeq Stranded Total RNA Library Prep Kit (Illumina, San Diego, CA), including a ribosomal RNA removal step. Sequencing was performed using the NovaSeq 6000 System (Illumina, San Diego, CA), and the obtained sequences were assembled using Geneious Prime (version 2024.0). The virus isolated in Chungcheongnam‐do was designated SNUVP2403A, and the virus isolated in Gyeongsangbuk‐do was designated SNUVP2407B.

### 2.6. Genetic and Phylogenetic Analysis

PRRSV‐2 whole genome sequences (*n* = 95, File S1), including SNUVP2403A and SNUVP2407B, were aligned using the MUSCLE algorithm. Phylogenetic trees based on ORF5 and whole genome sequences were constructed with RAxML using the GTRGAMMA model and 1000 bootstrap replicates. Phylogenetic trees were visualized with FigTree (v1.4.4).

Nucleotide sequence identities between SNUVP2403A, SNUVP2407B, and representative strains were analyzed. The representative strains included KU‐N1606, GBGJ22, CBJE19 (LKA); GGYC45, JB15‐N‐PJ10‐GN (LKB); K07−2273 (LKC); IA/2014/NADC34 (L1A); NADC30 (L1C); VR2332, RespPRRS_MLV (L5); and JXwn06 (L8). The similarities were assessed at the level of complete genome, nsps, and ORFs using MEGA11 software. In addition, the insertion and deletion patterns of nsp2 were analyzed in comparison with VR2332.

### 2.7. Recombination Analysis

Recombination analysis of SNUVP2403A and SNUVP2407B was performed using RDP4 (v4.101). The potential parental strains included KU‐N1606 (LKA), JB15‐N‐PJ10‐GN (LKB), K07−2273 (LKC), IA/2014/NADC34 (L1A), NADC30 (L1C), and RespPRRS_MLV (L5) [20], which were aligned using the MUSCLE algorithm. Recombination analysis was conducted by applying seven methods: RDP, GENECONV, BootScan, MaxChi, Chimaera, SiScan, and 3Seq. Recombination events were considered valid when it was supported by at least five out of the seven methods with a significance level of *p*  < 0.01 [23].

To visually verify recombination events and breakpoint positions, similarity plot analysis was conducted using SimPlot (v3.5.1). The window size was set to 500 bp and the step size to 20 bp. The final determination of recombination events was based on the combined results of RDP4 and SimPlot.

### 2.8. Animal Experiment Design

Thirty piglets at 3 weeks of age were purchased from a PRRSV‐free farm. All animals were confirmed negative for PRRSV, porcine circovirus type 2, and *Mycoplasma hyopneumoniae* by real‐time PCR. In addition, they were seronegative for PRRSV by ELISA. Six pigs were randomly assigned to each cage, and a total of five cages were utilized in the experiment. Two cages were assigned for the SNUVP2403A‐inoculated group (*n* = 12), two cages for the SNUVP2407B‐inoculated group (*n* = 12), and the remaining cage for the control group (*n* = 6).

After 3 days of acclimation, the SNUVP2403A‐inoculated group was intramuscularly inoculated with 2 mL of the SNUVP2403A strain (10^5^ TCID_50_/mL). The SNUVP2407B‐inoculated group was challenged in the same manner. The control group received an intramuscular injection of 2 mL of filtered PAM cell culture supernatant. Rectal temperature, respiratory signs, and clinical symptoms were monitored daily. Respiratory symptoms were scored according to the following criteria: 0 = normal, 1 = mild dyspnea upon handling, 2 = mild dyspnea at rest, 3 = moderate dyspnea upon handling, 4 = moderate dyspnea at rest, 5 = severe dyspnea upon handling, 6 = severe dyspnea at rest [24]. A rectal temperature above 40°C was considered a fever [25]. Body weight was measured at 0, 4, 7, 10, and 14 days post‐inoculation (dpi), and the average daily weight gain (ADWG) was calculated. Blood samples were collected from the jugular vein at 0, 4, 7, 10, and 14 dpi for quantification of PRRSV genomic copy numbers.

At 14 dpi, pigs were humanely euthanized by electrical stunning following intramuscular injection of azaperone (2 mg/kg, 1 mL, StressGuard, Dong Bang Inc., Seoul, Korea). Lesion evaluation was conducted by two pathologists blinded to group allocation. Tissue samples (100 mg each) from the lung, tonsil, thymus, mediastinal lymph node, spleen, heart, and liver were collected for RNA extraction. Lungs were also fixed in 10% neutral buffered formalin for histopathological examination. The animal experiment was conducted with the approval of the Institutional Animal Care and Use Committee of Seoul National University (IACUC, SNU‐250217‐6‐1).

### 2.9. Quantification of Viral Genomes and Antibody Response

RNA was extracted from serum and tissue homogenates using the phenol–chloroform method. The extracted RNA was quantified using the OneStep qRT‐PCR Master Mix (BioFACT, Daejeon, KR). The forward primer was 5′‐TCCAGATGCCGTTTGTGCTT‐3′ and the reverse primer was 5′‐GACGCCGGACGACAAATG‐3′ [26].

To establish standard curves, the ORF7 regions of SNUVP2403A and SNUVP2407B, which contain the target sequence for the primers, were cloned into the pBHA vector (Bioneer, Daejeon, Korea). The cycle threshold (Ct) values of serially diluted pBHA plasmids were obtained and then standard curves were generated. From the standard curves, formulas were derived to convert Ct values to Log_10_ RNA copies/mL [26–29].

Antibody responses in pigs were evaluated using a commercial enzyme‐linked immunosorbent assay kit (IDEXX PRRS X3 Ab Test; IDEXX, ME, USA). The assay was conducted in accordance with the manufacturer’s instructions, and samples with a sample‐to‐positive (S/P) ratio of ≥0.4 were interpreted as seropositive.

### 2.10. Pathologic Analysis

Macroscopic and microscopic lung lesions were blindly evaluated by two pathologists using the scoring system developed by Halbur et al. [24]. Gross lung lesions were assessed for each lung lobe and scored as the percentage of pneumonic area relative to the total lung volume. The total score was 100 points, with 27.5 points assigned to the right caudal and left caudal lobes each, 10 points each to the right cranial, right middle, cranial part of the left cranial lobe, and caudal part of the left cranial lobe, and 5 points to the accessory lobe.

Lung sections stained with hematoxylin and eosin were evaluated based on the severity of interstitial pneumonia. The scoring criteria were as follows: 0 = no lesions, 1 = mild interstitial pneumonia, 2 = moderate multifocal interstitial pneumonia, 3 = moderate diffuse interstitial pneumonia, 4 = severe interstitial pneumonia.

### 2.11. Immunohistochemistry for Detection of the PRRSV Antigen

The fixed tissues were embedded in paraffin to prepare tissue blocks. Sections were cut at a thickness of 4 μm using a microtome and mounted to adhesive slides. The slides underwent deparaffinization and hydration. Antigen retrieval was performed using Proteinase K (Invitrogen, Carlsbad, CA), and blocking was conducted with 10% normal goat serum. The slides were incubated overnight with the primary antibody SR‐30 that detects the highly conserved nucleocapsid protein of PRRSV. The goat anti‐mouse antibody conjugated with alkaline phosphatase (Thermo Fisher Scientific, Waltham, MA) was used as the secondary antibody. Signal detection was performed using Vector Red AP Substrate. Finally, the slides were counterstained with hematoxylin.

### 2.12. Statistical Analysis

Data analysis was conducted using IBM SPSS Statistics 29.0.1.0. The Kruskal–Wallis test was performed to compare the three groups: SNUVP2403A‐inoculated group, SNUVP2407B‐inoculated group and control group. A *p*‐value greater than 0.05 indicated no statistically significant difference among the groups. When the *p*‐value was less than 0.05, it indicated a significant difference between at least one pair of groups, and pairwise comparisons were subsequently performed by the Mann–Whitney test with Holm–Bonferroni correction.

## 3. Results

### 3.1. Isolation and Identification of PRRSV

IFA was performed on PAMs and MARC‐145 cells inoculated with the third passage viruses isolated from two farms. PRRSV nucleocapsid protein was detected in PAM cells, confirming that the two strains were successfully isolated (Figure 1a). Although viral isolation was attempted in MARC‐145 cells, PRRSV antigen was not detected in either strain.

Figure 1Isolation and growth kinetics of SNUVP2403A and SNUVP2407B in PAMs. (a) ICC revealed red staining of the PRRSV nucleocapsid protein. IFA showed nuclei (blue fluorescence) and the PRRSV nucleocapsid protein (green fluorescence). (b, c) PAMs were inoculated with the viruses at an MOI of 0.01, and culture supernatants were collected at 0, 12, 24, 36, and 48 hpi. Viral genomic copies were quantified by qRT‐PCR (b), and viral titers were determined by ICC (c). Data represent the mean ± standard deviation from three independent experiments.

**Figure:**
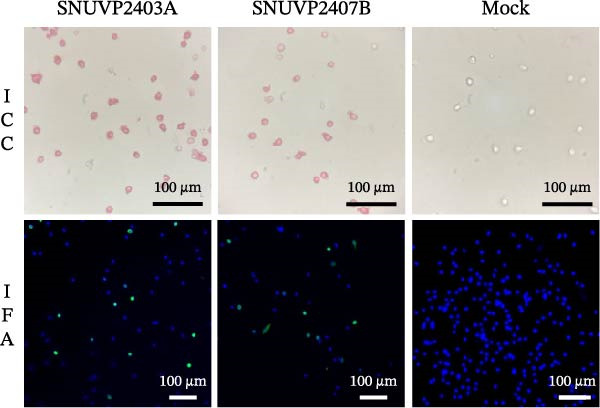
(a)

**Figure:**
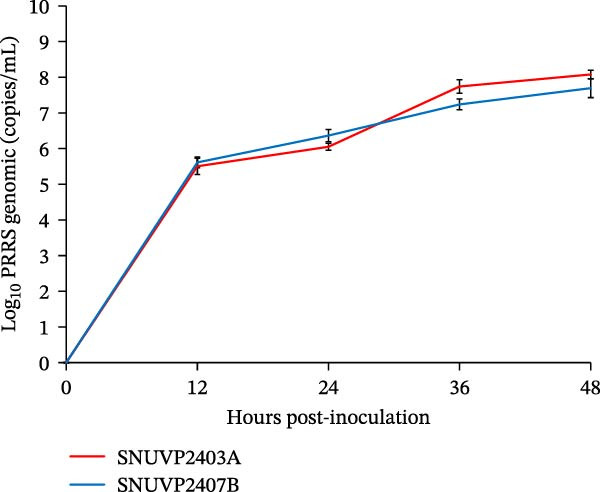
(b)

**Figure:**
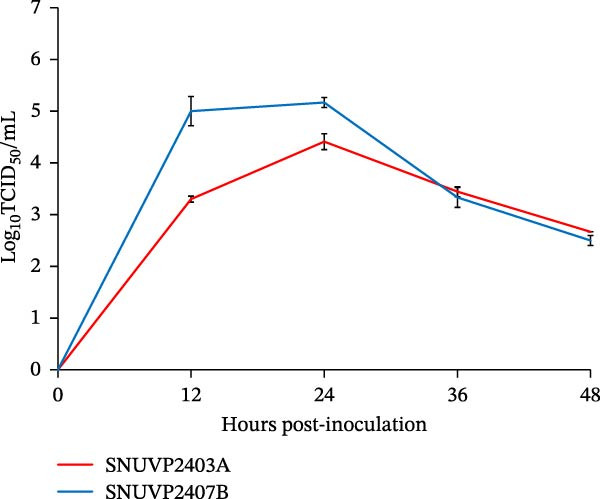
(c)

In PAM cells, genomic copy numbers of SNUVP2403A and SNUVP2407B exhibited a similar pattern (Figure 1b). The infectious titers of both strains peaked at 24 hpi, followed by a decline. In particular, SNUVP2407B rapidly produced infectious virus, reaching 10^5^ TCID_50_/mL at 12 hpi (Figure 1c).

### 3.2. Phylogenetic and Genetic Characterization

The complete genomes of the two strains were obtained, including the 5^′^ and 3^′^ UTRs but excluding the poly(A) tail. The genome length of SNUVP2403A was 15,156 bp, and that of SNUVP2407B was 14,951 bp. The whole genome sequences of the two strains were uploaded to NCBI (GenBank accession no. PX438683 for SNUVP2403A; GenBank accession no. PX438684 for SNUVP2407B). SNUVP2403A clustered with LKA strains in the phylogenetic tree based on the ORF5 sequences (Figure 2). At the whole genome level, SNUVP2403A exhibited the closest relationship to GBGJ22, with 87.9% sequence identity. The nucleotide sequences of nsp6 to nsp12 showed high similarity to RespPRRS_MLV (86%–97.9%) (Table 1).

Figure 2Phylogenetic analysis of SNUVP2403A and SNUVP2407B. (a) Phylogenetic tree based on the complete genome sequences. (b) Phylogenetic tree based on ORF5 sequences. LKA strains are labeled in red and LKB strains in blue. SNUVP2403A and SNUVP2407B are highlighted in yellow with red and blue borders, respectively. The green box indicates NADC30‐like strains clustered with SNUVP2407B. Both phylogenetic trees were generated using the RAxML program with the GTRGAMMA model and 1000 bootstrap replicates.

**Figure:**
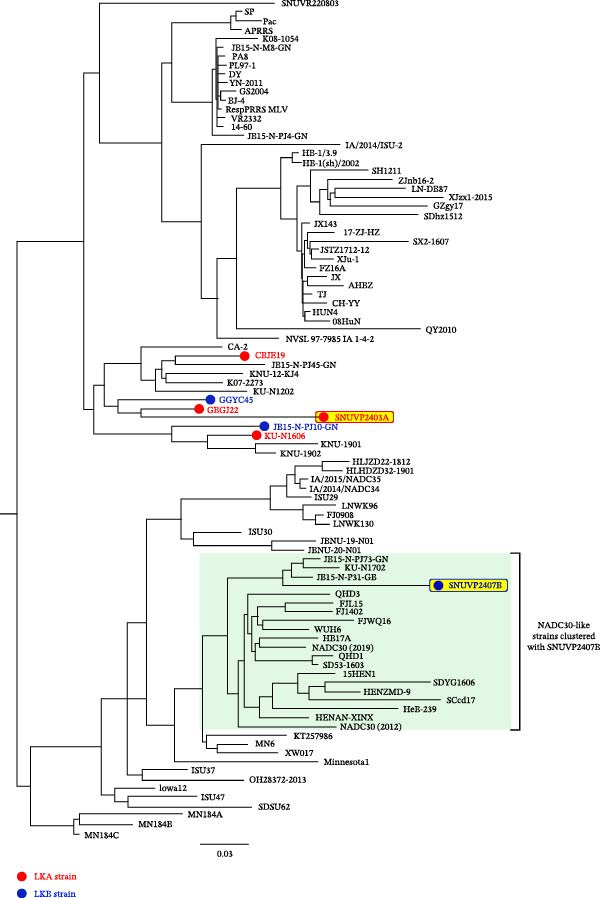
(a)

**Figure:**
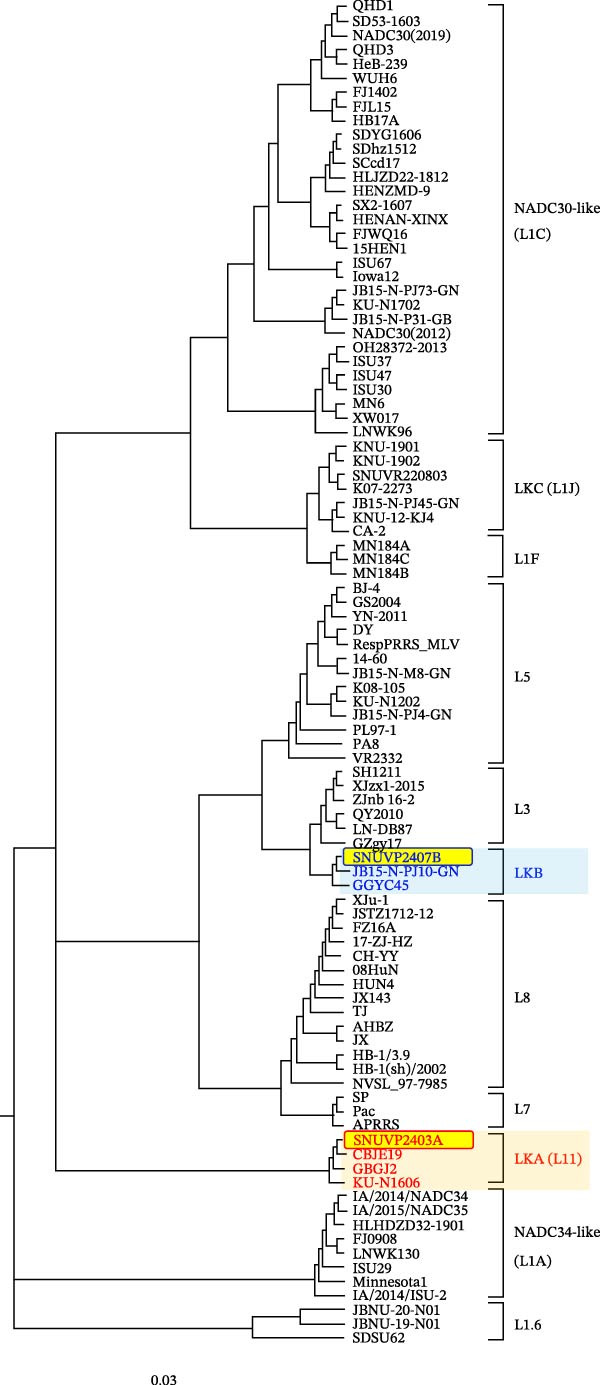
(b)

**Table 1:** Genetic identity between SNUVP2403A and representative strains of PRRSV‐2.

Genomic regions	Nucleotide identity with SNUVP2403A (%)										
	LKA			LKB		LKC	L1A	L1C	L5		L8
	MK057530	MZ287315	MZ287316	MZ287324	MZ287321	MZ287326	MF326985	JN654469	EF536003	AF066183	EF641008
	KU‐N1606	GBGJ22	CBJE19	GGYC45	JB15‐N‐PJ10‐GN	K07‐2273	IA/2014/NADC34	NADC30	VR2332	Ingelvac MLV (RespPRRS_MLV)	JXwn06
WGS	82.8	**87.9**	83.5	85.7	81.8	84	81	79.2	84.7	85	80.7
ORF1	81.4	**87.5**	82.6	85.9	80.6	84.1	77.8	80.2	79.2	84.2	84
nsp1	78.7	86.7	82.5	**87**	76.6	85.1	79.5	81.9	82.3	82.2	80.1
nsp2	77.6	**81.5**	79.3	79.7	76.9	81	69.5	74	69.2	69.4	64.5
nsp3	85.6	**89**	85.7	87.1	86.5	87.6	80.1	80	81.1	81.5	78.9
nsp4	83.7	**86.5**	83.6	85.8	84.1	86.4	78	76.3	77.7	78.2	81.1
nsp5	76.7	82.3	78.7	**85.5**	72.2	82.8	69	74.3	71.9	71.9	72.1
nsp6	79.6	**90.9**	88.4	88.4	82.6	85.8	91	88.4	**90.9**	**90.9**	85.8
nsp7	77	**87**	78	80	75.4	77.9	74.9	78.1	85.4	86	77.8
nsp8	84	89.5	76.6	89.6	80.7	78.8	**90.3**	85.9	89.5	89.5	88.5
nsp9	93	96.7	92.2	96.2	92.4	92.5	92.9	93.3	**97.9**	**97.9**	94.8
nsp10	85.5	93	87.6	90.5	85.2	88.7	82	83	94.6	**94.8**	86.7
nsp11	82.9	89.3	84.6	87.2	82.1	86.6	82.2	86.2	92	**92.3**	85
nsp12	81.9	88.7	84.2	87.7	82.6	84	79.6	85.3	**89.8**	**89.8**	84.5
ORF2	87.3	**88.3**	85.9	87.5	85.4	84.7	83.6	83.7	86.6	87.1	87.3
ORF3	87.1	**88.2**	81.4	84.3	85.9	81.4	79	79.7	85.6	85.7	84.5
ORF4	88.3	**90.9**	81.2	87	86.8	81.1	82	82.7	87.2	87.2	84.8
ORF5	87.2	**89.6**	88.8	81.4	84.5	85	85.8	84	85.3	85.3	83.4
ORF6	87.6	**92**	91.4	88.9	90.2	87	85.1	84.8	90.5	90.2	88.4
ORF7	86.2	88.3	**89.4**	84.6	82	86	85	85.3	87.3	87.7	84.4

*Note:* Percentages with the highest identity to SNUVP2403A are shown in bold.

SNUVP2407B formed a distinct cluster with LKB strains in the phylogenetic tree based on ORF5 sequences and was classified as LKB (Figure 2). In the phylogenetic tree based on the whole genome sequences, SNUVP2407B clustered with NADC30‐like strains. SNUVP2407B exhibited high nucleotide sequence similarity to NADC30 from nsp1 to nsp10 (86.9%–95.8%), and to JB15‐N‐PJ10‐GN from nsp11 to ORF7 (88.8%–93.5%) (Table 2).

**Table 2:** Genetic identity between SNUVP2407B and representative strains of PRRSV‐2.

Genomic regions	Nucleotide identity with SNUVP2407B (%)										
	LKA			LKB		LKC	L1A	L1C	L5		L8
	MK057530	MZ287315	MZ287316	MZ287324	MZ287321	MZ287326	MF326985	JN654469	EF536003	AF066183	EF641008
	KU‐N1606	GBGJ22	CBJE19	GGYC45	JB15‐N‐PJ10‐GN	K07‐2273	IA/2014/NADC34	NADC30	VR2332	Ingelvac MLV (RespPRRS_MLV)	JXwn06
WGS	79.6	79.5	79.2	79.8	81.3	79.7	79.1	**87.5**	78.6	81.2	81
ORF1	78.3	77.7	77.7	78.1	78	78.2	78	**88.6**	76.4	79.3	79.2
nsp1	77.3	80.1	77	80.4	76.5	80.7	80.1	**89.1**	79.4	79.7	80.1
nsp2	69.8	70.4	72.4	72.1	69.7	73	68.5	**86.9**	68.8	68.9	64.6
nsp3	79.6	78.8	77.9	77.9	77.7	79.8	83.5	**91.3**	82.1	82.2	81.7
nsp4	78.9	77	78.1	77.7	79.5	76.7	78.8	**92.3**	80.9	81	80.5
nsp5	75.8	75.3	74.4	78.5	73.2	76.6	77.5	**90.7**	83.3	83.3	82.3
nsp6	82.6	93.4	90.9	90.9	85.5	88.5	88.5	**95.7**	93.4	93.4	91
nsp7	73.9	74.5	79.4	78.9	74.2	79	76.5	**88.9**	76.4	77	77
nsp8	84.9	80.7	73.1	81.9	82.6	75.5	86.7	**89.5**	83.7	83.7	83.7
nsp9	90.8	90.9	90	90.8	90.4	89.2	92.3	**95.8**	91.8	91.8	91.8
nsp10	84.7	82.7	83.7	80.9	84.7	83.7	83	**88**	84	84	84.2
nsp11	**88.8**	83.8	80.6	82.3	**88.8**	82.3	81.6	83.1	85.7	85.7	85.9
nsp12	92.1	83.4	81.9	80.1	**93.2**	79.3	76.6	83.8	85.3	85.3	84.7
ORF2	85.1	85.1	86.1	85.4	**90.9**	83.3	81	81.8	85.2	86	85.4
ORF3	83	84.9	83.3	82.1	**90**	85.2	81.6	82	85.9	85.9	85.7
ORF4	86.2	86.2	81.5	87.6	**90.7**	85.2	83.1	85	87.1	87.1	87.5
ORF5	78.6	80.1	78.6	80.5	**91.2**	81.7	80.5	78.6	81.9	81.9	80.3
ORF6	86.4	89.1	89.1	90.5	91.9	86.7	85.6	86.8	**92.3**	92.1	92.1
ORF7	88.6	85.9	86.4	86.1	**93.5**	91.3	84.2	86.6	90.9	91.3	91.6

*Note:* Percentages with the highest identity to SNUVP2407B are shown in bold.

**Figure 3:**
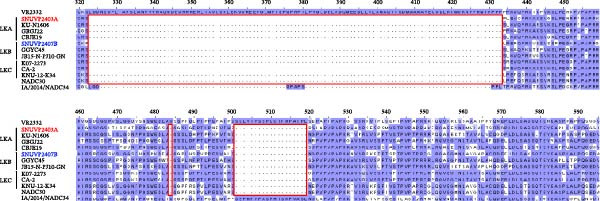
nsp2 amino acid alignment. The red box represents the amino acid deletion (111 + 1 + 19) of LKA, LKB, LKC, and NADC30 strains based on the VR2332 strain.

The nsp2 regions of SNUVP2403A and SNUVP2407B contained a discontinuous 131‐amino acid deletion (111 + 1 + 19) based on the reference strain VR2332. All other LKA, LKB, and LKC strains showed the same deletion pattern, which is identical to NADC30 strain (Figure 3).

### 3.3. Recombination Analysis

RDP4 analysis indicated that no recombination events were detected between SNUVP2403A and any of the six potential parental strains. Recombination analysis of SNUVP2403A with 93 strains (File S1) was conducted, but recombination signal was not detected. In contrast, a recombination event was identified in SNUVP2407B, with NADC30 as the major parent and JB15‐N‐PJ10‐GN as the minor parent. The recombination event was supported by all detection methods (Table 3). The recombination breakpoint was located in the nsp10 region, with the 1–9899 nt region derived from NADC30 and the 9900–14,951 nt region derived from JB15‐N‐PJ10‐GN. SimPlot analysis was conducted using SNUVP2407B as the query and NADC30 and JB15‐N‐PJ10‐GN as the parental strains. It was confirmed that the recombination event was located in the nsp10 region (Figure 4a). Consistent with the recombination analysis, phylogenetic analysis showed that SNUVP2407B clustered with NADC30 in the 1–9899 nt region and with JB15‐N‐PJ10‐GN in the 9900–14,951 nt region (Figure 4b).

Figure 4Recombination analysis of SNUVP2407B. (a) The recombination event of the SNUVP2407B strain was visualized using SimPlot software. NADC30 and JB15‐N‐PJ10‐GN (LKB) were used as parental strains based on the results of RDP4. The recombination breakpoint is located within the nsp10 region and is indicated by a dashed line. (b) Phylogenetic trees were constructed based on the recombination breakpoint of SNUVP2407B.

**Figure:**
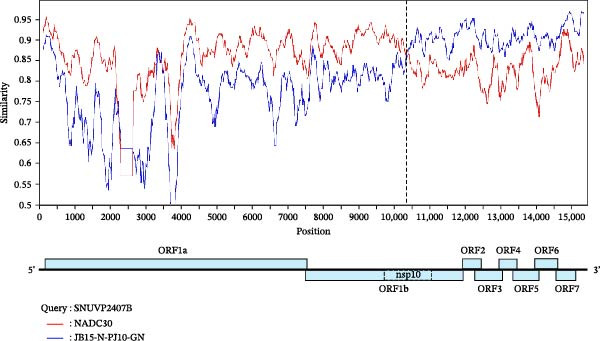
(a)

**Figure:**
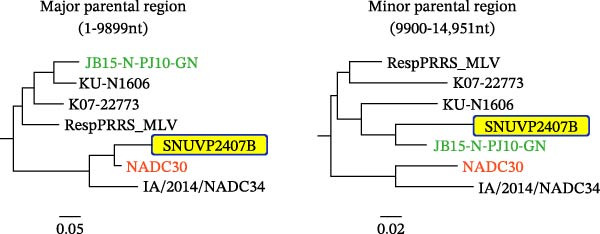
(b)

**Table 3:** Recombination event of SNUVP2407B detected by RDP4.

Query	Parental strain		*p*‐Value of the recombination analysis method						
	Major	Minor	RDP	GENECONV	BootScan	MaxChi	Chimaera	SiScan	3Seq
SNUVP2407B	NADC30 (1–9899 nt)	JB15‐N‐PJ10‐GN (9900–14,951 nt)	4.902 × 10^−45^	6.161 × 10^−3^	3.953 × 10^−39^	2.506 × 10^−28^	8.243 × 10^−32^	3.223 × 10^−30^	1.165 × 10^−14^

### 3.4. Clinical Observation

**Figure 5:**
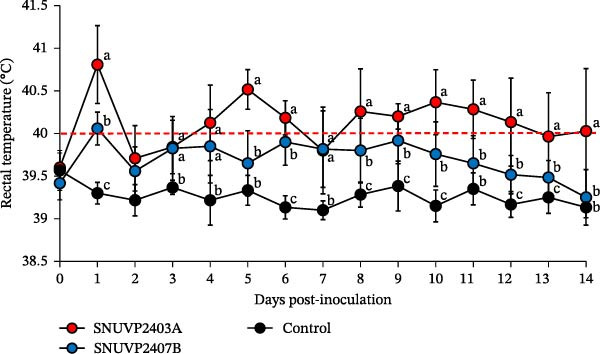
Rectal temperature. The red dashed line indicates the fever threshold. The significantly different groups (*p* < 0.05) are marked with alphabets.

The pigs inoculated with SNUVP2403A and SNUVP2407B showed elevated body temperatures at 1 dpi, which then decreased at 2 dpi. In the SNUVP2403A‐inoculated group, body temperature peaked at 40.51°C at 5 dpi and fever (≥40°C) was observed for 10 days during the experiment. In contrast, the SNUVP2407B‐inoculated pigs did not show any fever after 1 dpi and returned to normal body temperature at 14 dpi with no significant difference compared with the control group. The control group maintained a normal body temperature ranging from 39.1°C to 39.5°C (Figure 5).

The group inoculated with SNUVP2403A showed significantly lower ADWG than the control group throughout the entire experimental period. The SNUVP2407B‐inoculated pigs showed significantly reduced weight gain compared with the control group during 0–4 and 7–10 dpi. However, the ADWG of the SNUVP2407B group was not significantly different from the control group during 10–14 and 0–14 dpi, indicating recovery to a normal growth rate (Table 4).

**Table 4:** Body weight, ADWG, and lung gross/microscopic lesion scores of the SNUVP2403A and SNUVP2407B‐inoculated and control groups.

Measures	dpi	SNUVP2403A	SNUVP2407B	Control
Body weight (kg)	0	6.44 ± 0.38	6.48 ± 0.63	6.28 ± 0.17
	4	7.03 ± 0.41^a,b^	6.98 ± 0.98^b^	7.68 ± 0.3^a^
	7	7.53 ± 0.49^b^	7.88 ± 1.14^ab^	8.48 ± 0.44^a^
	10	8.12 ± 0.85^b^	8.57 ± 1.29^a,b^	9.6 ± 0.43^a^
	14	9.00 ± 1.18^b^	10.16 ± 1.47^a,b^	11.48 ± 0.44^a^
ADWG (g/pig/day)	0–4	157.5 ± 83.37^b^	122.92 ± 117.48^c^	350.00 ± 101.24^a^
	4–7	183.33 ± 63.34^b^	300 ± 184.23^a^	266.67 ± 73.03^a^
	7–10	166.71 ± 202.41^b^	230.56 ± 166.03^b^	372.22 ± 38.97^a^
	10–14	183.33 ± 236.51^b^	397.92 ± 135.45^a^	470.83 ± 65.99^a^
	0–14	183.12 ± 93.8^b^	262.5 ± 69.19^a,b^	371.43 ± 36.14^a^
Lung gross lesion score	14	24.82 ± 7.81^a^	11.83 ± 7.37^b^	0^c^
Lung microscopic lesion score	14	1.72 ± 0.8^a^	1.18 ± 0.36^b^	0^c^

*Note:* Different letters indicate statistically significant differences (*p*  < 0.05).

**Figure 6:**
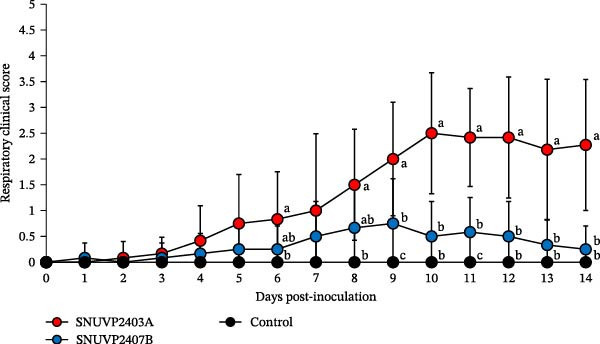
Respiratory clinical scores. The significantly different groups (*p* < 0.05) are marked with alphabets.

The control group did not exhibit respiratory signs or other clinical signs. The SNUVP2403A‐inoculated pigs experienced progressively worsening respiratory symptoms until 10 dpi, which persisted through 14 dpi (Figure 6). Nasal discharge, coughing, eyelid edema, and cyanosis were observed (Figure 7). One pig was found dead at 13 dpi, and interstitial pneumonia was confirmed upon necropsy. The SNUVP2407B‐inoculated group experienced only mild respiratory symptoms, with no significant difference from the control group (Figure 6). Six pigs had mild dyspnea with clinical scores ranging from 1 to 2, while the remaining six pigs did not develop any respiratory symptoms. No other clinical signs or death were observed in the SNUVP2407B‐inoculated group.

Figure 7Clinical signs observed in pigs infected with SNUVP2403A. (a) Nasal discharge. (b) Eyelid edema.

**Figure:**
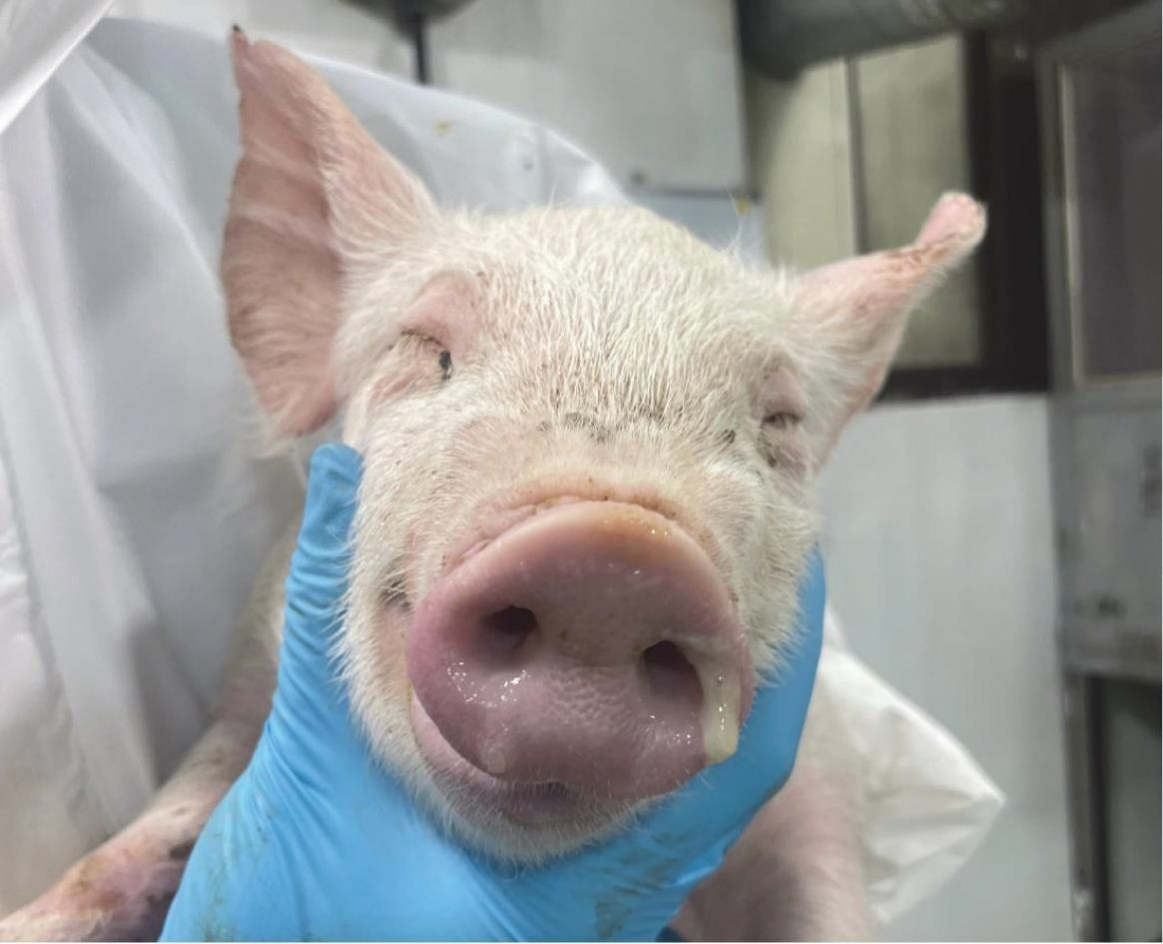
(a)

**Figure:**
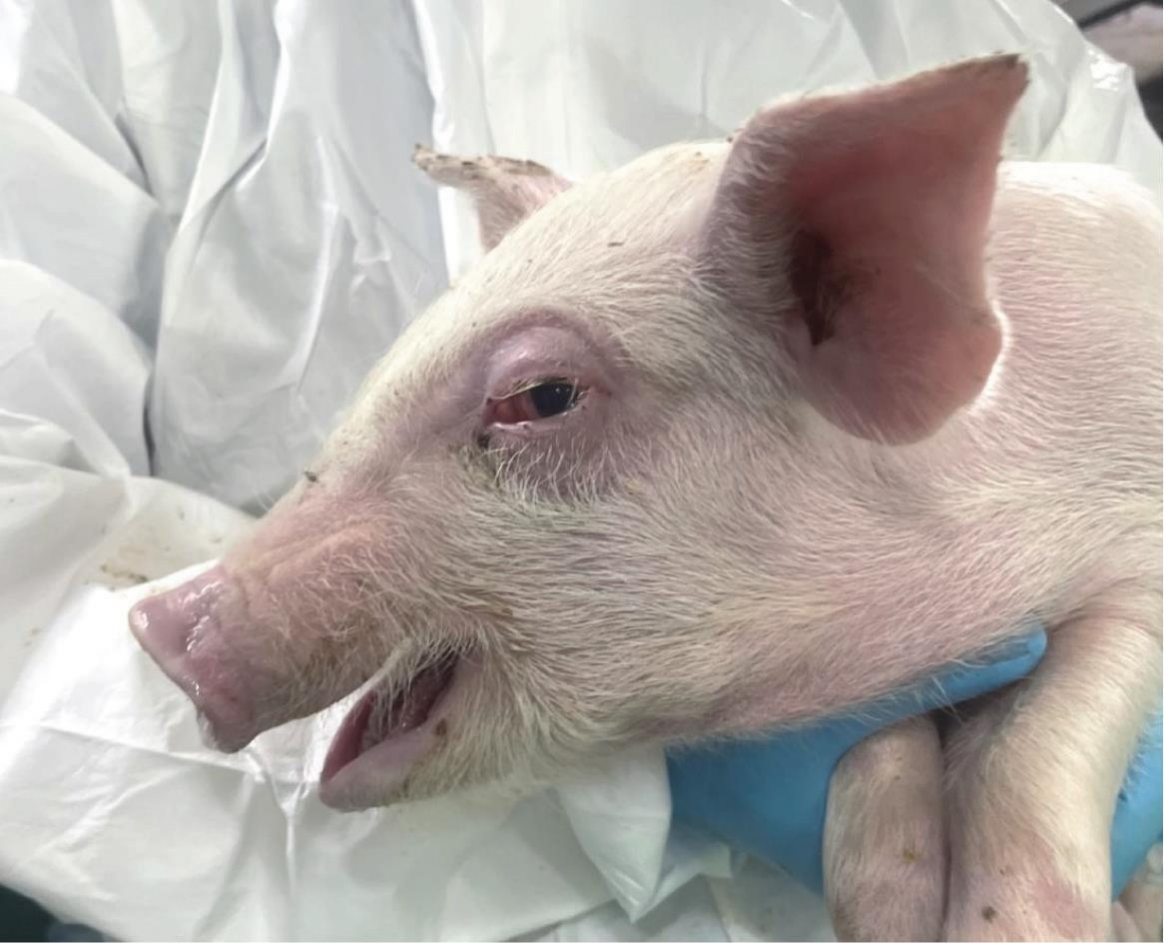
(b)

### 3.5. PRRSV Quantification and Antibody Response

In the SNUVP2403A‐inoculated group, the serum viral load peaked at 10^6.7^ genomic copies/mL at 7 dpi and then gradually decreased. The viremia in the SNUVP2407B‐inoculated group reached 10^5.9^ genomic copies/mL at 4 dpi and rapidly declined at 7 dpi. From 7 to 14 dpi, viremia of the SNUVP2407B‐inoculated group persisted at a low level (10^2.3^–10^2.7^ genomic copies/mL) (Figure 8a). PRRSV genomic copies were detected in all organs collected from both inoculated groups. There was no significant difference in the viral RNA loads of the lung, tonsil, thymus, lymph node, and liver between the SNUVP2403A and SNUVP2407B‐inoculated groups. However, the viral RNA loads in the heart and spleen were significantly higher in the SNUVP2403A‐inoculated group than in the SNUVP2407B‐inoculated group (Figure 8b). In the control group, no PRRSV genomic copies were detected in any serum or tissue samples.

Figure 8(a) Viral load detected in serum by qRT‐PCR. (b) Viral load detected in various organs collected at necropsy by qRT‐PCR. (c) PRRSV‐specific antibody levels expressed as S/P ratios. The dashed line indicates the positive cutoff value (S/P = 0.4). The significantly different groups (*p* < 0.05) are marked with alphabets.

**Figure:**
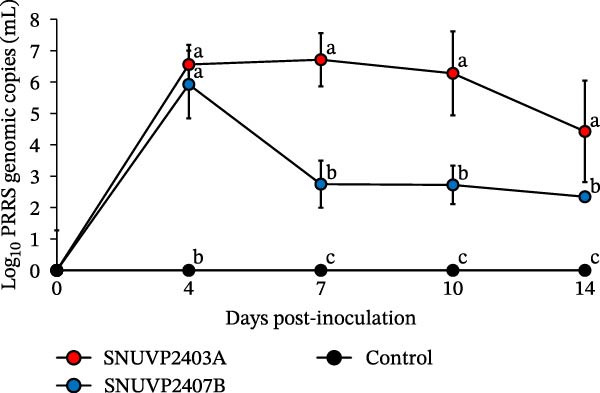
(a)

**Figure:**
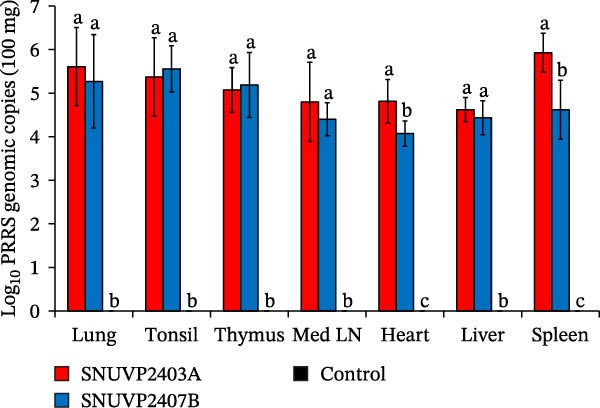
(b)

**Figure:**
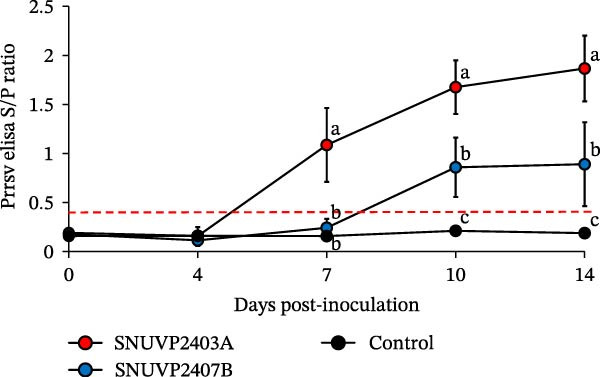
(c)

Seroconversion in the SNUVP2403A‐inoculated group began at 7 dpi. In contrast, seroconversion in the SNUVP2407B‐inoculated group was first detected at 10 dpi, and the S/P ratios from 7 to 14 dpi were significantly lower than those in the SNUVP2403A‐inoculated group. Notably, no seroconversion was observed in the SNUVP2407B‐inoculated group at 7 dpi (Figure 8c), indicating that the rapid decline in viral load was not attributable to antibody responses.

### 3.6. Pathology and Immunohistochemistry

All pigs, except for one that died at 13 dpi, were humanely euthanized at 14 dpi. SNUVP2403A and SNUVP2407B‐inoculated pigs exhibited interstitial pneumonia characterized by lung consolidation and failure to collapse. Gross lung lesions were assessed based on the extent of interstitial pneumonia, and SNUVP2403A‐inoculated pigs had significantly higher scores compared with SNUVP2407B‐inoculated pigs (Figure 9a).

Figure 9Gross lesions, histopathology, and IHC of the lungs from pigs at 14 dpi. (a) SNUVP2403A and SNUVP2407B‐inoculated pigs exhibited interstitial pneumonia with lung consolidation and failure to collapse. Histopathology revealed thickening of the alveolar septa due to inflammatory cell infiltration and epithelial hyperplasia. The SNUVP2403A‐inoculated group showed significantly higher interstitial pneumonia scores, both gross and microscopic, compared with the SNUVP2407B‐inoculated group. (b) IHC confirmed PRRSV antigen in the lungs of both inoculated groups, with red signals detected in macrophages within the alveolar septa.

**Figure:**
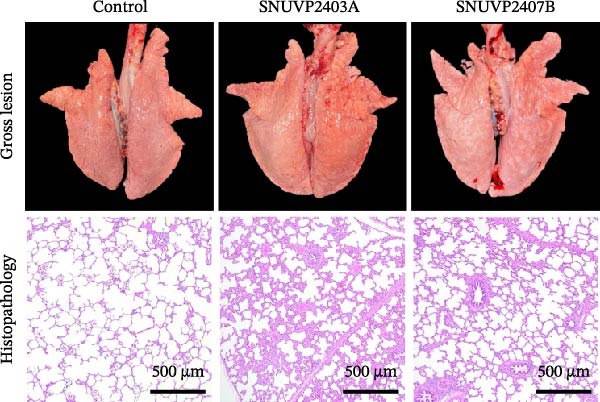
(a)

**Figure:**
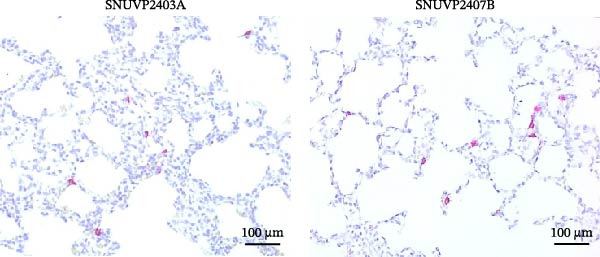
(b)

The control group exhibited normal pulmonary architecture, characterized by thin alveolar septa lined with a single layer of epithelial cells. In contrast, both SNUVP2403A and SNUVP2407B‐inoculated groups showed thickening of the alveolar septa due to inflammatory cell infiltration and type II pneumocyte hyperplasia. The microscopic lung lesion score was significantly higher in SNUVP2403A‐inoculated pigs compared with SNUVP2407B‐inoculated pigs (Table 4). Pigs in the SNUVP2403A‐inoculated group exhibited moderate multifocal interstitial pneumonia with reduced alveolar spaces, whereas the SNUVP2407B‐inoculated group showed mild interstitial pneumonia (Figure 9a).

Immunohistochemistry confirmed the presence of PRRSV antigens in the lungs of pigs inoculated with SNUVP2403A and SNUVP2407B. Red signals were detected in macrophages infiltrating the alveolar septa (Figure 9b).

## 4. Discussion

In this study, we isolated a non‐recombinant LKA strain, SNUVP2403A, and a naturally recombinant LKB variant, SNUVP2407B. SNUVP2403A showed pathogenicity comparable to previously reported LKA strains. However, studies on CBJE19 and GBGJ22 were conducted in 4‐week‐old pigs using lower challenge doses [19]. Considering these differences in experimental conditions, our findings indicate similar or lower pathogenicity of SNUVP2403A compared with CBJE19 and GBGJ22. In contrast, pigs inoculated with SNUVP2407B exhibited only mild respiratory symptoms, and all animals survived throughout the experiment. These results contrast with previous reports in which LKB strains exhibited 25%–100% mortality [19].

SNUVP2403A showed high nucleotide sequence similarity to RespPRRS_MLV in the nsp8 to nsp12 region. This finding is consistent with a previous report that GBGJ22 was recombined with RespPRRS_MLV in the same genomic region [20]. However, no recombination event was detected in SNUVP2403A. This can be explained by the fact that frequent mutation accumulation in RNA viruses can make it difficult to detect past recombination events [30, 31]. Accordingly, it is possible that the ancestor of SNUVP2403A possessed a similar recombination pattern to GBGJ22, but accumulation of mutations increased genetic diversity, making the recombination event undetectable.

Genetic analysis revealed that SNUVP2407B is a recombinant derived from NADC30 and JB15‐N‐PJ10‐GN. This finding represents a novel recombination pattern within the LKB lineage, indicating the potential for genetic variation in LKB strains. Furthermore, SNUVP2407B exhibited the highest genetic similarity to JB15‐N‐PJ10‐GN from the nsp11 to ORF7 region. Given that recent LKB isolates are genetically similar to JB15‐N‐PJ10‐GN [32], SNUVP2407B reflects this current epidemiological trend.

Recombination is considered a major determinant of PRRSV pathogenicity [33, 34] and may be associated with the relatively low virulence of SNUVP2407B. Previous studies have investigated which genomic regions of PRRSV are associated with attenuation. For example, a chimeric virus lost its pathogenicity when the 5′UTR–ORF1 region of highly pathogenic MN184 was replaced with that of RespPRRS_MLV [35]. Similarly, SNUVP2407B has a comparable recombination pattern, and its pathogenicity was attenuated compared with the parental strain, JB15‐N‐PJ10‐GN. These findings suggest that the nsp regions of NADC30 may contribute to the reduced pathogenicity of SNUVP2407B. However, further studies are required to determine which specific genomic regions contribute to the low pathogenicity of SNUVP2407B.

Recombinant PRRSV strains tend to show pathogenicity within the range of their parental strains [36, 37]. However, several exceptions have been reported. For example, SNUVR220803 displayed higher virulence than its parental strains, K07−2273, and RespPRRS_MLV [21]. Conversely, SDVD‐NMG2023, a recombinant of NADC30, NADC34, and JXA1, showed attenuated pathogenicity relative to its parental strains [38]. Similarly, SNUVP2407B exhibited reduced pathogenicity compared with its parental strains, suggesting that the pathogenicity of certain PRRSV strains may fall outside the range of the parental strains.

During PRRSV infection, viremia is typically sustained during the acute phase, with viral loads peaking around 7–10 dpi and subsequently declining over time [39, 40]. The SNUVP2403A‐inoculated group exhibited a typical viremia pattern. In contrast, the SNUVP2407B‐inoculated group showed an early peak at 4 dpi, followed by a decline. Similar viremia kinetics have also been reported for other PRRSV‐2 strains. For example, P129 and JA142 strains reached peak viremia at 4 and 5 dpi, respectively, and subsequently decreased [41, 42]. Notably, P129 showed no significant differences in body weight gain or lung lesion scores compared with the control group [41] and JA142 strain exhibited moderate virulence but low mortality [43]. Therefore, further studies are required to investigate the association between this viremia pattern and low to moderate virulence.

In viral replication, it has been reported that rapid replication rather than high fidelity provides immediate fitness under selection pressure [44–46]. SNUVP2407B produced infectious virus more rapidly than SNUVP2403A in vitro. The rapid replication of SNUVP2407B may have conferred a selective advantage despite attenuated pathogenicity. Previous studies have shown inconsistent associations between replication speed and pathogenicity in PRRSV; high replication efficiency in PAM cells was associated with the virulence of HP‐PRRSV [47], whereas no such association was observed for NADC34‐like PRRSV‐2 [48]. Thus, further research is needed to clarify the relationship between the replication efficiency of SNUVP2407B in vitro and its pathogenicity. Notably, the recombination breakpoint of SNUVP2407B is located in nsp10, which regulates the replication rate in vivo and in vitro [47, 49]. Therefore, the nsp10 region where recombination occurred may have influenced the rapid replication of SNUVP2407B.

A primary limitation of this study is that only a single isolate from each lineage was analyzed. In addition, differences in field pathogenicity were observed between two farms indicating that the viruses were derived from specific outbreak situations. Despite these limitations, this study provides a systematic characterization of two field isolates and improves our understanding of LKA and LKB. In particular, the unique recombination pattern and the viremia kinetics of SNUVP2407B provide insights into low‐virulence PRRSV. Moreover, these findings highlight substantial pathogenic heterogeneity among the lineages and suggest the need for continuous surveillance.

## 5. Conclusion

SNUVP2403A was classified as LKA and SNUVP2407B as LKB. SNUVP2403A showed no detectable recombination and exhibited pathogenicity similar to or lower than previously reported LKA strains. In contrast, SNUVP2407B was identified as a recombinant between NADC30 and JB15‐N‐PJ10‐GN, with a breakpoint located in nsp10, and showed markedly attenuated pathogenicity compared with LKB strains isolated before 2015. These findings provide insights into the genetic and pathogenic diversity of LKA and LKB.

## Funding

The author’s research was supported by the Research Institute for Veterinary Science, College of Veterinary Medicine, Seoul National University (Grant 550‐20220119), and by the BK21 FOUR Future Veterinary Medicine Leading Education and Research Center.

## Ethics Statement

All experimental protocols were approved prior to the study by the Seoul National University Institutional Animal Care and Use Committee (IACUC, SNU‐250217‐6‐1).

## Conflicts of Interest

The authors declare no conflicts of interest.

## Supporting Information

Additional supporting information can be found online in the Supporting Information section.

## Supporting information


**Supporting Information** List of 95 PRRSV‐2 strains used for phylogenetic tree construction. The table includes the GenBank accession numbers, strain names, lineage classifications, and years of collection for all strains.

## Data Availability

The data that support the findings of this study are available from the corresponding author upon reasonable request.
